# Digital ethnographic analysis of prostate cancer discussions on social media

**DOI:** 10.1002/bco2.64

**Published:** 2020-12-31

**Authors:** Sriram V. Eleswarapu, Tommy Jiang, Jesse N. Mills, Vadim Osadchiy

**Affiliations:** ^1^ Division of Andrology Department of Urology David Geffen School of Medicine University of California Los Angeles CA USA; ^2^ Consortium for Health Activity on Social Media David Geffen School of Medicine University of California Los Angeles CA USA

## 1 INTRODUCTION

Men struggling with prostate cancer report significant decision‐related distress surrounding cancer surveillance and treatment.^1^, ^2^ Typically, prostate cancer shared decision making (SDM) involves three main parties: the patient, patient’s loved one(s), and physician. However, individuals now increasingly turn to online resources to inform these decisions,^3^ though this has been less extensively studied.

Online discussion boards offer discussants anonymity that facilitates conversations without potential embarrassment. Similarly, patients have the option of speaking directly with others who may be further along in their cancer treatment, allowing for unfiltered feedback related to personal experiences and struggles. Previously published studies exploring online discussion boards about similarly sensitive topics, such as erectile dysfunction, have revealed valuable insights into the concerns and anxieties of discussants.^4^

Here, we employ a mixed‐method approach to understand online prostate cancer discussions and how they may impact patient’s decision making. In leveraging both quantitative natural language processing (NLP)‐based approaches alongside manual post‐annotation, we reveal the important role of social media in prostate cancer decision making and concerns of patients at different points along the treatment spectrum.

We selected four publicly available online discussion boards with the greatest number of prostate cancer posts from January through December 2019 using the search criteria “prostate cancer discussion board” and “prostate cancer forum” in an incognito Google Chrome browser without geographic or language restrictions: CancerResearchUK.org (249 posts), CSN.Cancer.org/Forum (158), HealthBoards.com (72), and CancerCompass.com/Message‐Board.htm (27). All posts including the term “prostate” were extracted. Posts were defined as the initial discussant’s body of text in a discussion thread. Response posts that were part of the same thread were excluded.

We performed a NLP analytic technique called the Meaning Extraction Method with principal component analysis (MEM/PCA)^5^ on all extracted posts (step‐by‐step explanation in Jiang et al^4^). Once word clusters were grouped, a common descriptive theme was manually assigned to each cluster by mutual agreement of the first and senior author. We further interrogated a subset of 145 posts for quantitative data on whether relevant topics of discussion were mentioned, including quality of life (QOL) concerns (eg, erectile dysfunction, bowel or bladder incontinence), longevity discussions (eg, prognosis), therapeutic modalities (eg, prostatectomy, radiation therapy), and active surveillance. We also collected information on the author of each post (patient vs family/caregiver) and whether a post was authored prior to or after any therapeutic intervention.

Chi‐squared and Fisher exact tests were used to compare categorical variables, and negative binomial regression was used to compare count variables. Multivariate logistic regression was used to identify predictors of discussions of QOL or longevity. RStudio 1.1.463 was used for statistical analysis, with p<0.05 considered statistically significant.

We extracted a total of 506 posts from the online discussion boards. MEM/PCA on all posts revealed word clusters that organized into the following themes: *Diagnostics*, *Treatment Decisions*, *Quality of Life Considerations*, *Treatment Planning*, *Biopsy Results*, and *Patient‐Physician Interaction*. Words and their respective factor loadings that define each theme are seen in Table 1. Selected sample excerpts highlighting these themes are also included. Demographic data are shown in Table S1.

**TABLE 1:** Quantitative thematic analysis results. Six thematic clusters and associated factor loading coefficients, thresholded at 0.30, derived from the meaning extraction method with principal component analysis. Excerpts from posts highlighting each theme have also been included. Abbreviations: FL, factor loading

Themes (Factor loading score)			Sample quotes
*Diagnostics*			
Biopsy (0.57) MRI (0.54) PSA (0.53) Test (0.53) Urologist (0.48) DRE (0.46) Result (0.45)	Blood (0.41) Show (0.39) Enlarge (0.38) Concern (0.37) History (0.36) High (0.35) Week (0.34)	Normal (0.34) Doctor (0.33) Level (0.32) Order (0.32) Worry (0.31) Thought (0.30) Area (0.30)	“If an MRI is the be all/end all in determining prostate cancer [...] why do I need a biopsy” “I am wondering if anyone on the forum had his prostate removed following an MRI i.e. without a biopsy of the prostate ever having been completed?”
*Treatment decisions*			
Surgery (0.49) Prostatectomy (0.47) Offer (0.43) Call (0.42) Radical (0.40) Surgeon (0.40) Low (0.40)	Treatment (0.39) Continue (0.38) Option (0.38) Patient (0.37) Consider (0.36) Radiation (0.36) Decide (0.32)	Treat (0.31) Man (0.31) Place (0.31) Experience (0.31)	“He did say that I could have a prostatectomy or radiotherapy if I choose, each would be a high success rate, but he is not sure that dealing with the side effects of those methods would outweigh holding off on any treatment and just doing the active surveillance […] Any thoughts, experiences, or anything else anyone can share would be greatly appreciated.” “There is a part of me saying, ‘hey, this is cancer, don't mess around with it’. The other part is wondering if this is an unnecessary procedure at this point in time.”
*Quality of life considerations*			
Love (0.50) Care (0.47) Day (0.42) Better (0.38) Life (0.37) Hard (0.37) Work (0.37)	Time (0.36) Die (0.34) Family (0.33) See (0.33) Change (0.32) Best (0.32) Issue (0.32)	Live (0.32) Turn (0.31) Point (0.31) Under (0.30)	“Did you experience urine leakage, and if so how much and how long did it last for. Did you have any Bowel side effects and if so, what were they and have they died off?” “You need to seriously consider your options [...] I almost went the radiation route, but was warned about the potential aftereffects. My friend may end up, permanently, with a urine bag that goes directly to his bladder; that he will have to empty for the remainder of his life.”
*Treatment planning*			
Hormone (0.60) Lymph (0.56) Chemo (0.55) Node (0.52) Bone (0.50) Start (0.44) Oncologist (0.44)	Therapy (0.40) Scan (0.36) Injection (0.35) Advance (0.34) Month (0.32) CT (0.32)		“My father in law has advanced prostate cancer. It has spread to his liver, bones, lungs & found out today that his lymph nodes have wrapped around his kidneys. We were told that this is what would kill him. Does anyone know roughly how long he might have left?”
*Biopsy results*			
Core (0.57) Gleason (0.56) Positive (0.55) Grade (0.44) Active (0.40) Score (0.40) Opinion (0.39)	Right (0.36) Recommend (0.35) Total (0.32) Left (0.31)		“The doctor told me only one core sample had cancer, and the amount was very very small. We decided to do active surveillance. Fast forward to now. My PSA has gone up to 19 […] Does the increased PSA necessarily mean that the cancer has grown/spread?”
*Patient‐physician interaction*			
Spread (0.49) Dad (0.48) Hospital (0.41) Lung (0.38) Effect (0.37) Side (0.37) Nurse (0.34)	Prostate (0.34) Told (0.33) Cancer (0.32) Appointment (0.31)		“The Urologists explanation was it is standard procedure and would be malpractice to not do it, then he basically dismissed my follow‐up questions. Doesn't give me the warm fuzzies about the necessity of this procedure.” “The consultant is so harsh, and doesn’t mix his words you see. Quote 'zytiga ok for a year or two, chemo ok for 18 months' it does leave you feeling very worried, and adding up the time he has got.”

Multivariate comparisons revealed that posts were more likely to feature discussions of QOL factors if they were authored by the patient (OR: 2.10, *P *= 0.049) and if they were written after initiating a therapeutic intervention (OR: 2.56, *P *= 0.027) (Table S2). Posts that featured discussions surrounding life expectancy and prognosis were more likely to mention more than two therapeutic interventions (OR: 4.41, *P *= 0.020) and less likely to have that post authored by the patient himself (OR: 0.39, *P *= 0.012) (Table S3).

With the recent growth of readily available cancer‐related information online, discussions that occur outside of the clinic play an increasingly valuable role in decision making. Our results reveal that many patients struggle with a different facet of decision making during each aspect of their clinical course including diagnosis, treatment, and posttreatment care. Using NLP and multivariate analysis, we sought to better characterize when QOL factors and life expectancy may be pertinent concerns to a patient in his prostate cancer care and how these are integrated into the decision‐making process.

Results from our thematic analysis highlight the substantial anxiety that comes with a diagnosis of prostate cancer. Our data suggest that anxiety surrounding decision making may be a key driver for this distress, as many discussants expressed extreme discomfort in deciding between a therapeutic intervention and active surveillance, or between weighing QOL factors and cancer cure in deciding on a therapeutic intervention. It is important to underscore, though, that the act of sharing these concerns online may be therapeutic. These patients may, as a result, be more likely to make decisions related to prostate cancer treatment that more closely align with their goals.

Patients were more likely than partners or family members to emphasize QOL factors, though these discussions were often featured *after* a therapeutic intervention had already been initiated. Online discussion boards also create a space for caregivers of patients with prostate cancer to discuss their collective experiences and unique challenges. Our data reveal that patients were more likely than their partners or caregivers to emphasize QOL issues online. In contrast, patients’ caregivers were primarily concerned with life expectancy as it related to selection of treatment modalities. This is consistent with data from Reamer et al., who showed that patients with prostate cancer who consult with their support network were over 11 times more likely to choose a treatment based on life expectancy and cure rather than treatment‐related QOL effects.^6^

Several posts highlighted complementary medicine as an appealing option within the context of treatment decision making. These alternative interventions were often framed as having efficacy without any negative impact on QOL. Previous work has reported that, unfortunately, inaccurate information proliferates online, especially related to urological conditions, including prostate cancer. Additionally, patients may also have trouble finding and evaluating the vast amount information readily available online.^7^, ^8^

Our study is not without limitations. We were unable to present information related to age, cancer risk stratification or other data points that would assist in contextualizing our findings. Posts represent a snapshot in time, limiting long‐term follow‐up decisions or a standardized measure of concerns between discussion posts. In identifying discussion forums for analysis, we used the search engine Google; factors such as geography and search engine optimization may have impacted forum selection. Nearly 50% of the analyzed posts were from a United Kingdom‐based online discussion board; these discussions may not reflect the SDM process of patients from other countries as therapeutic options available in those countries may differ. Additionally, individuals who seek information online through discussion boards may be different with respect to information preferences compared to those who do not turn to the internet for similar concerns.

Online discussion boards represent an important space where patients and caregivers can discuss shared experiences surrounding all aspects of their prostate cancer experience. By analyzing unfiltered social media data, we have the opportunity to truly listen to our patients; our findings can be used to better inform decision aid development, as best practices often recommend developing such tools by initially querying patients. This study also suggests a potential role for physicians on social media to engage directly with patients online and connect them to accurate resources.

## COMPETING INTEREST

Sriram Eleswarapu is a consultant for Metuchen Pharmaceuticals. Jesse Mills is a consultant for Antares Pharma, Boston Scientific, and Endo Pharmaceuticals. Tommy Jiang and Vadim Osadchiy have no disclosures.

## Funding information

Sriram Eleswarapu is supported by a Research Scholar Award from the Urology Care Foundation and the American Urological Association

## Supporting information

Table S1

Table S2

Table S3

## References

[1] Davison BJ , So AI , Goldenberg SL . Quality of life, sexual function and decisional regret at 1 year after surgical treatment for localized prostate cancer. BJU Int. 2007;100:780–5. 17578466 10.1111/j.1464-410X.2007.07043.x

[2] Steginga SK , Occhipinti S , Gardiner RA , Yaxley J , Heathcote P . Prospective study of men's psychological and decision‐related adjustment after treatment for localized prostate cancer. Urology. 2004;63:751–6. 15072894 10.1016/j.urology.2003.11.017

[3] Bender JL , Feldman‐Stewart D , Tong C , Lee K , Brundage M , Pai H , et al. Health‐related internet use among men with prostate cancer in Canada: cancer registry survey study. J Med Internet Res. 2019;19(21):e14241. 10.2196/14241 PMC6891399 31742561

[4] Jiang T , Osadchiy V , Mills JN , Eleswarapu SV . Is it all in my head? Self‐reported psychogenic erectile dysfunction and depression are common among young men seeking advice on social media. Urology. 2020;142:133–40. 32437776 10.1016/j.urology.2020.04.100

[5] Chung C , Pennebaker J . Revealing dimensions of thinking in open‐ended self‐descriptions: An automated meaning extraction method for natural language. J Res Pers. 2008;42:96–132. 18802499 10.1016/j.jrp.2007.04.006 PMC2542899

[6] Reamer E , Yang F , Holmes‐Rovner M , Liu J , Xu J . Influence of men's personality and social support on treatment decision‐making for localized prostate cancer. Biomed Res Int. 2017;2017:1467056. 28785574 10.1155/2017/1467056 PMC5529637

[7] Alsyouf M , Stokes P , Hur D , Amasyali A , Ruckle H , Hu B . “Fake News” in urology: evaluating the accuracy of articles shared on social media in genitourinary malignancies. BJU Int. 2019;124:701–6. 10.1111/bju.14787 31044493

[8] Zaila KE , Osadchiy V , Shahinyan RH , Mills JN , Eleswarapu SV . Social media sensationalism in the male infertility space: a mixed methodology analysis. World J Mens Health. 2020;38. 10.5534/wjmh.200009 PMC7502321 32378368

