# Supplementary material for: Digital ethnographic analysis of prostate cancer discussions on social media
Source: BJUI Compass. 2020 Dec 31;2(2):82–5. doi: 10.1002/bco2.64 (PMC8988692; doi:10.1002/bco2.64)
Supplement: Supplementary file 1 — Table S1 [file BCO2-2-82-s002.docx]

| **Characteristic** | **Total**  **(n = 145)** | **QOL Discussed**  **(n = 57)** | **QOL Not Discussed**  **(n = 88)** | **p** | **Life Expectancy Discussed**  **(n = 54)** | **Life Expectancy Not Discussed**  **(n = 91)** | **p** |
| --- | --- | --- | --- | --- | --- | --- | --- |
| Authored by Patient | 79 (54%) | 37 (65%) | 42 (48%) | **0.042** | 21(39%) | 58 (64%) | **0.004** |
| Authored After Therapeutic Intervention Started | 56 (39%) | 29 (51%) | 27 (31%) | **0.015** | 17 (31%) | 39 (43%) | 0.174 |
| Quantity of Therapeutic Interventions Mentioned | 1.13 **±** 1.04 | 1.30 **±** 1.10 | 1.02 **±** 0.99 | 0.225 | 1.30 **±** 1.19 | 1.03 **±** 0.94 | 0.243 |
| Prostatectomy Mentioned | 42 (29%) | 18 (32%) | 24 (27%) | 0.577 | 14 (26%) | 28 (31%) | 0.534 |
| ADT Mentioned | 42 (29%) | 20 (35%) | 22 (25%) | 0.191 | 20 (37%) | 22 (24%) | 0.099 |
| Radiation Therapy Mentioned | 49 (34%) | 23 (40%) | 26 (30%) | 0.179 | 22 (41%) | 27 (30%) | 0.173 |
| Brachytherapy Mentioned | 7 (5%) | 4 (7.0%) | 3 (3.4%) | 0.434 | 2 (3.7%) | 5 (5.5%) | 1.000 |
| Chemotherapy Mentioned | 24 (17%) | 9 (16%) | 15 (17%) | 0.842 | 12 (22%) | 12 (13%) | 0.157 |
| Active Surveillance Discussions Mentioned | 7 (4.8%) | 1 (1.8%) | 6 (6.8%) | 0.246 | 2 (3.7%) | 5 (5.5%) | 1.000 |
| Life Expectancy Discussions Mentioned | 54 (37%) | 18 (32%) | 36 (41%) | 0.256 | - | - | - |
| QOL Discussions Mentioned | 57 (39%) | - | - | - | 18 (33%) | 39 (43%) | 0.256 |

**Supplemental Table 1. Post Characteristics.** Abbreviations: ADT, androgen deprivation therapy; QOL, quality of life.
