# Supplementary material for: Digital ethnographic analysis of prostate cancer discussions on social media
Source: BJUI Compass. 2020 Dec 31;2(2):82–5. doi: 10.1002/bco2.64 (PMC8988692; doi:10.1002/bco2.64)
Supplement: Supplementary file 2 — Table S2 [file BCO2-2-82-s003.docx]

**Supplemental Table 2. Multivariate Predictors of Quality of Life Discussions.**

| **Predictor** | **OR** | **p** | **95% CI** |
| --- | --- | --- | --- |
| **Author** |  |  |  |
| Partner or Family | Ref |  |  |
| Patient | 2.10 | **0.049** | 1.01-4.47 |
| **Timeline** |  |  |  |
| Prior to Therapeutic Intervention | Ref |  |  |
| After Therapeutic Intervention | 2.56 | **0.027** | 1.13-6.02 |
| **Quantity of Therapeutic**  **Interventions Mentioned** |  |  |  |
| 0 | Ref |  |  |
| 1-2 | 0.64 | 0.329 | 0.25-1.56 |
| >2 | 1.52 | 0.519 | 0.42-5.46 |
| **Active Surveillance Mentioned** | 0.17 | 0.118 | 0.01-1.13 |
| **Life Expectancy Mentioned** | 0.76 | 0.492 | 0.35-1.65 |
