# Supplementary material for: Digital ethnographic analysis of prostate cancer discussions on social media
Source: BJUI Compass. 2020 Dec 31;2(2):82–5. doi: 10.1002/bco2.64 (PMC8988692; doi:10.1002/bco2.64)
Supplement: Supplementary file 3 — Table S3 [file BCO2-2-82-s001.docx]

**Supplemental Table 3. Multivariate Predictors of Life Expectancy Discussions.**

| **Predictor** | **OR** | **p** | **95% CI** |
| --- | --- | --- | --- |
| **Author** |  |  |  |
| Partner or Family | Ref |  |  |
| Patient | 0.39 | **0.012** | 0.18-0.80 |
| **Timeline** |  |  |  |
| Prior to Therapeutic Intervention | Ref |  |  |
| After Therapeutic Intervention | 0.56 | 0.188 | 0.24-1.32 |
| **Quantity of Therapeutic**  **Interventions Mentioned** |  |  |  |
| 0 | Ref |  |  |
| 1-2 | 1.36 | 0.494 | 0.56-3.35 |
| >2 | 4.41 | **0.020** | 1.31-16.30 |
| **Active Surveillance Mentioned** | 0.57 | 0.571 | 0.35-1.65 |
| **Quality of Life Factors Mentioned** | 0.76 | 0.489 | 0.06-3.48 |
